# Continuous glucose monitoring for detection of glycemic variability, hypoglycemia, and hyperglycemia in women with eating disorders

**DOI:** 10.1186/s13030-022-00251-4

**Published:** 2022-10-27

**Authors:** Nao Uotani, Shun’ichi Noma, Momoko Akamine, Takashi Miyawaki

**Affiliations:** 1 Graduate School of Home Economics, Department of Living Environment, Food and Nutrition, Kyoto Women’s University, 35 Kitahiyoshi-Cho, Imakumano, Higashiyama Kyoto, 605-8501 Japan; 2 Noma-Kokoro Clinic, 5-322-1 Sujikaibashi, Fukakusa, Fushimi, Kyoto, 612-0889 Japan

**Keywords:** Intermittently scanned continuous glucose monitoring system, Mean amplitude of glycemic excursions, Glycemic variability, Hypoglycemia, Hyperglycemia, Anorexia nervosa, Bulimia nervosa, Eating disorder

## Abstract

**Background:**

The aim of this study was to investigate the relationships between hypoglycemia, hyperglycemia, glycemic variability (GV), and eating behavior by measuring daily glucose levels through an intermittently scanned continuous glucose monitoring (isCGM) system in outpatients classified according to eating disorder subtypes.

**Methods:**

We analyzed data for 18 patients (four ANR, nine ANBP, and five BN cases). A FreeStyle Libre Pro® device was attached to the posterior aspect of the upper arm for glucose monitoring. This device conducted measurements every 15 min for five consecutive days. We estimated the mean amplitude of glycemic excursions (MAGE), hypoglycemia, and hyperglycemia.

**Results:**

The mean glucose levels were 91.1 ± 2.2 mg/dL in the ANR group, 94.8 ± 7.5 mg/dL in the ANBP group, and 87.1 ± 8.0 mg/dL in the BN group (*P* = 0.174). The overall mean MAGE index was 52.8 ± 20.5 mg/dL. The mean MAGE values according to the subtypes were 42.2 ± 5.6 mg/dL in the ANR group, 57.4 ± 23.7 mg/dL in the ANBP group, and 53.0 ± 21.8 mg/dL in the BN group (*P* = 0.496). Over the course of five days, the frequency of hypoglycemia was as follows: three occurrences in the ANBP group, five occurrences in the BN group, and no occurrences in the ANR group (*P* = 0.016). Moreover, the occurrence of hypoglycemia was statistically significantly higher in the BN group than in the ANR group (*P* = 0.013). In the BN group, the frequency of hypoglycemia was highest between 2 and 6 AM, while hypoglycemia was observed throughout the day in the ANBP group. The frequency of hyperglycemia was one occurrence in the ANR group, one occurrence in the BN group, and zero occurrences in the ANBP group (*P* = 0.641).

**Conclusions:**

Varying GV, hypoglycemia, and hyperglycemia were observed in all subtypes of eating disorders. Our findings suggest that eating behaviors such as binge eating and purging are associated with GV and hypoglycemia. We showed the importance of developing different nutritional approaches tailored to the subtype of eating disorder to prevent hypoglycemia. Additional studies are needed to explore the relationship between glucose levels and eating behaviors in patients with eating disorders.

## Background

Patients with eating disorders present with abnormal eating behaviors, including extreme eating restrictions, binge eating and purging, excessive fear of obesity, and distortion of body image (i.e., body dysmorphia). According to the 5th edition of the Diagnostic and Statistical Manual of Mental Disorders (DSM-5, American Psychiatric Association, 2013), eating disorders are classified into anorexia nervosa (AN), bulimia nervosa (BN), binge eating disorder, and other specified subtypes of feeding or eating disorders [1]. Eating disorders are associated with various mental and physical comorbidities and can cause severe morbidity and premature death. Starvation, electrolyte disturbances, dehydration, an increased risk of suicide, and alcoholism have been reported as direct or indirect causes of mortality in patients with eating disorders [2]. The mortality rate for patients with AN is 1.36–20%, which is one of the highest mortality rates for any mental disorder [3].

Previous studies have reported that patients with eating disorders often have abnormally low or abnormally high blood glucose levels, as shown below. Hypoglycemia often occurs in patients with AN who have abnormal eating behaviors, such as eating restrictions, and is considered one of the causes of sudden death in patients with AN [3]. Meanwhile, patients with BN are reported to be 2.4 times more likely than controls of the same healthy age to develop type 2 diabetes due to repeated binge eating [4]. Therefore, it is important to understand more thoroughly the characteristics of hypoglycemia and hyperglycemia in patients with eating disorders, as this can inform effective prevention and intervention efforts. However, to our knowledge, there are no detailed reports on the development of hypoglycemia and hyperglycemia among patients with eating disorders classified into subtypes.

The importance of monitoring glycemic variability (GV) using continuous glucose monitoring (CGM) systems has been investigated in recent years [5, 6]. More specifically, GV refers to changes in blood glucose levels. GV also has a broader meaning, in that it alludes to glucose fluctuations that occur throughout the day, including hypoglycemic periods and postprandial increases [5]. The mean amplitude of the glycemic excursion (MAGE) is an indicator of GV. Within CGM, MAGE represents the mean value of glucose fluctuations that exceeds 1 standard deviation as obtained from 24-h glucose fluctuations, as well as the range of diurnal glucose fluctuations. Therefore, MAGE can estimate sharp increases and falls in glucose levels, and this metric is designed to quantify fluctuations in glucose levels without reference to mean glucose levels. Some studies have reported the average reference range for MAGE indices representing normal glucose tolerance is 30–40 mg/dL [7–9].

Recently, CGM systems and intermittently scanned continuous glucose monitoring (isCGM) systems, which continuously measure glucose levels in interstitial fluid, have been used in the treatment of patients with diabetes who require strict glycemic control [10]. FreeStyle Libre Pro ® (Abbott, Chicago, IL, USA) is an isCGM system that can continuously measure glucose levels in tissue interstitial fluid with a microneedle in the center of the sensor after easily and painlessly attaching the sensor to the extension side of the upper arm. The sensor is 35 mm in diameter, 5 mm thick, and weighs 5 g. The resistance to water is verified for up to 30 min at a depth of 1 m. Patients can go about their daily lives without any inconvenience while wearing the sensor. The sensor automatically records glucose levels every 15 min and stores measurement data for up to 14 days without calibration during use. In this study, the data were read using a dedicated reader. Due to its simplicity, the isCGM system provides continuous glycemic control and has been widely implemented to detect hypoglycemia, hyperglycemia, and glucose fluctuations.

The aim of this study was to investigate the relationship between hypoglycemia, hyperglycemia, GV, and eating behaviors using the isCGM system by measuring daily glucose levels of outpatients classified into subtypes of eating disorders.

## Methods

**Fig. 1:**
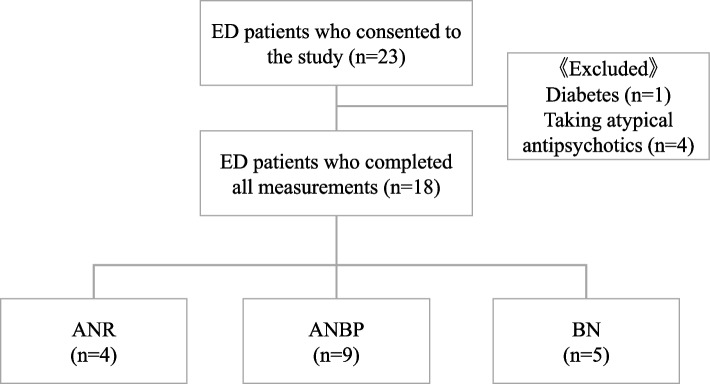
Participant selection criteria. ANR: anorexia nervosa, restricting subtype; ANBP: anorexia nervosa, binge eating/purging subtype; BN: bulimia nervosa

This is a cross-sectional study conducted from October 1, 2020 to September 30, 2021. We recruited 23 female patients with eating disorders who were undergoing outpatient treatment in a psychiatric clinic in Kyoto, Japan. The patients were diagnosed with AN (ANR, restricting subtype; or ANBP, binge-eating/purging subtype) or BN by a board-certified psychiatrist according to the DSM-5 criteria [1]. Figure 1 shows the study selection criteria. The study exclusion criteria were a diagnosis of diabetes and taking atypical antipsychotics that might affect glycemic fluctuation, such as multi-acting receptor-targeted antipsychotics, serotonin-dopamine antagonists, and dopamine system stabilizers. None of the participants were engaged in night shift work. We enrolled and analyzed data for 18 patients classified into ANR subtype (*n* = 4), ANBP subtype (*n* = 9), and BN subtype (*n* = 5).

### Glucose monitoring

A FreeStyle Libre Pro® device was affixed behind the upper arm of all patients presenting at the clinic. Monitoring was conducted every 15 min for 7 to 14 consecutive days. The measured data were extracted using a dedicated reader. Data analysis was performed using five consecutive days of measurement data (i.e., from the third day to the seventh day after the sensor was attached to maintain consistency). Mean glucose levels and MAGE indices were calculated at 24 h using a computer software associated with the monitoring device, Easy GV version 9.0. This computer-based software is available free of charge for non-commercial purposes by contacting N.R. Hill at the University of Oxford (Oxford, UK) [11].

The frequencies of hypoglycemia (defined as a glucose level < 60 mg/dL) and hyperglycemia (defined as a glucose level of ≥ 180 mg/dL) observed during the five-day monitoring period were also recorded. A cutoff glucose level of 60 mg/dL normally activates the inverse regulatory system, and patients without diabetes begin to experience the neuroglycemic and adrenergic symptoms characteristic of hypoglycemia [12, 13]. Using these data, absolute glucose levels as well as glucose fluctuations were compared according to the subtype of eating disorder.

### Daily records and the frequency of binge eating and purging

The patients kept daily records during the sensor monitoring period. This included their bedtimes and waking times, as well as daily dietary records (meal durations, binge-eating episodes, vomiting episodes) for three nonconsecutive days during the standard monitoring period. The frequency of purging (binge eating, vomiting, laxative abuse, compulsive exercise) during the last 28 days was also investigated.

Fasting venous blood samples were collected from all participants to assess baseline plasma glucose, serum insulin, and hemoglobin A1c (HbA1c) levels (i.e., glucose levels and fluctuation-related factors) when installing the isCGM sensor. Furthermore, as an indicator of insulin resistance, homeostasis model assessment of insulin resistance (HOMA-IR) was calculated as [(serum insulin in µU/mL) × (plasma glucose in mg/dL)/405] [14]. Pancreatic β-cell function was assessed using a homeostasis model assessment β-cell (HOMA-β) and calculated as [ (serum insulin in μU/mL × 360)/(plasma glucose in mg/dL-63)] [15].

### Anthropometric measurements

Anthropometric measurements were made along with biochemical measurements. Body weight was measured to the nearest 0.1 kg using an HD-661 digital health meter (Tanita, Tokyo, Japan) and height was measured to the nearest 0.1 cm using a metal height meter (Yoshida Co., Ltd., YS-OA). Body mass index (BMI) was calculated as body weight (kg) divided by height squared (m^2^).

### Statistical analyses

Data are shown as means ± standard deviations or as medians and interquartile ranges calculated according to the Shapiro–Wilk normality test. Statistical analyses were performed using Statistical Package for the Social Sciences statistical software (version 27.0 Windows-based software, IBM, Armonk, NY, USA). A one-way analysis of variance was implemented to compare the mean values between the three independent groups, and Tukey's honestly significant difference test was used to conduct multiple comparisons. The Kruskal–Wallis test was used to compare the median values between the three independent groups, and the Bonferroni correction was used to adjust for multiple comparisons. Fisher's exact test was used to compare the frequency statistics for categorical variables between the groups. The Spearman's rank correlation test was used to test the correlation between the frequency of hypoglycemia and body weight, and the correlation between glucose monitoring results and the frequency of binge eating and purging. The level of statistical significance was established at *P* < 0.05.

### Ethics considerations

All participants provided their written informed consent following an explanation of the study objectives and protocols. The study protocol was approved by the Ethics Committee of Kyoto Women’s University (ethics approval number 2019–37) and was conducted in accordance with the principles of the Declaration of Helsinki.

## Results

Table 1 shows the baseline information of the participants enrolled according to the subtype of eating disorders. There were no statistically significant differences in age or duration of the disease between the groups. There were no statistically significant differences between the groups in terms of baseline plasma blood glucose, serum insulin, HOMA-IR, HOMA-β and HbA1c levels.

**Table 1:** Baseline information

		All patients	①ANR	②ANBP	③BN	*P*	Multiple comparison
Number		18	4	9	5		
Age	year	31(26,37)	26(22,32)	31(29,33)	35(32,41)	0.298	
Disease duration	year	13.5 ± 12.4	4.5 ± 3.8	16.1 ± 14.3	16.8 ± 10.9	0.233	
Height	cm	158.9(155.0,162.2)	155.3(150.9,160.6)	157.4(155.2,160.0)	162.2(159.5,166.1)	0.290	
Weight	kg	42.3 ± 8.5	34.3 ± 5.8	40.1 ± 4.8	52.6 ± 5.1	** < 0.001**	①vs③*P* < 0.001, ②vs③*P* = 0.001
BMI	kg/m^2^	16.6 ± 2.6	14.1 ± 1.6	15.9 ± 1.8	19.9 ± 0.9	** < 0.001**	①vs③*P* < 0.001, ②vs③*P* = 0.001
The frequency of binge eating	times/28 days	-	0.0 ± 0.0	27.7 ± 21.1	6.4 ± 10.5	**0.021**	➀vs②*P* = 0.033
The frequency of vomiting	times/28 days	-	0.0 ± 0.0	31.0 ± 22.8	1.0 ± 1.0	**0.006**	➀vs②*P* = 0.019, ②vs③*P* = 0.015
The frequency of laxative abuse	times/28 days	-	0.0 ± 0.0	15.6 ± 24.6	9.0 ± 12.6	0.414	
The frequency of compulsive exercise	times/28 days	-	0.5 ± 1.0	3.4 ± 9.3	11.6 ± 13.8	0.224	
Plasma blood glucose	mg/dl	80.2 ± 10.6	79.0 ± 6.5	79.4 ± 13.6	83.0 ± 6.8	0.828	
Serum inslin	μU/ml	2.5(1.6,3.3)	2.8(1.6,4.1)	2.5(1.2,2.9)	2.4(2.3,3.9)	0.724	
HOMA-IR		0.52(0.25,0.69)	0.53(0.29,0.84)	0.52(0.22,0.66)	0.52(0.47,0.82)	0.674	
HOMA-β		43.2(29.7,82.8)	71.7(47.0,78.5)	41.5(27.2,90.0)	43.6(34.6,63.8)	0.840	
HbA1c	%	5.1 ± 0.2	5.3 ± 0.3	5.0 ± 0.2	5.2 ± 0.2	0.185	

Presented by mean ± SD or median (Q1,Q3) depending on the normality based on the Shapiro–Wilk test

*P* value was based on either One-way ANOVA or Kruskal–Wallis test depending on the normality based on the Shapiro–Wilk test

The multiple comparisons shown in the table are based on Tukey's HSD test

*Abbreviations*: *ED* Eating disorder, *ANR* Anorexia nervosa restricting, *ANBP* Anorexia nervosa binge eating/purging, *BN* Bulimia nervosa, *HOMA-IR* Homeostasis model assessment-insulin resistance, *HOMA-β* Homeostasis model assessment-beta cell

Table 2 shows the mean glucose levels, MAGE indices, and frequency of hypoglycemia and hyperglycemia occurrence during the glucose monitoring period of five days. The mean glucose level for all patients was 91.8 ± 7.3 mg/dL. The mean glucose levels by eating disorder subtype were 91.1 ± 2.2 mg/dL in the ANR group, 94.8 ± 7.5 mg/dL in the ANBP group, and 87.1 ± 8.0 mg/dL in the BN group. The mean MAGE index for all patients was 52.8 ± 20.5 mg/dL. The MAGE indices by eating disorder subtype were 42.2 ± 5.6 mg/dL in the ANR group, 57.4 ± 23.7 mg/dL in the ANBP group, and 53.0 ± 21.8 mg/dL in the BN group. No statistically significant differences were observed between the groups.

**Table 2:** The five-day glucose monitoring result

		All patients	①ANR	②ANBP	③BN	*P*	Multiple comparison
Number		18	4	9	5		
Mean glucose level	mg/dl	91.8 ± 7.3	91.1 ± 2.2	94.8 ± 7.5	87.1 ± 8.0	0.174	
MAGE	mg/dl	52.8 ± 20.5	42.2 ± 5.6	57.4 ± 23.7	53.0 ± 21.8	0.496	
The frequency of hypoglycemia occurrence for the five-day (glucose < 60 mg/dl)	occurrences/5 days	3(0,12)	0(0,0)	3(1,12)	5(3,29)	**0.016**	①vs③*P* = 0.013
The proportions of patients with hypoglycemia	number (%)	12(67%)	0(0%)	7(78%)	5(100%)	0.005 *	
The frequency of hyperglycemia occurrence for the five-day (glucose > = 180 mg/dl)	occurrences/5 days	0(0,3)	1(0,2)	0(0,1)	1(0,5)	0.641	
The proportions of patients with hyperglycemia	number (%)	8(44%)	2(50%)	3(33%)	3(60%)	0.827 *	

Presented by mean ± SD or median (Q1,Q3) depending on the normality based on the Shapiro–Wilk test

P value was based on either One-way ANOVA, Kruskal–Wallis test or Fisher's exact test* depending on the normality based on the Shapiro–Wilk test

The multiple comparisons shown in the table are based on the Bonferroni test

*Abbreviations ED* Eating disorder, *ANR* Anorexia nervosa restricting, *ANBP* Anorexia nervosa binge eating/purging, *BN* bulimia nervosa, *MAGE* Mean amplitude of glycemic excursions

The frequency of hypoglycemia occurrence for the five-day monitoring period was as follows: three occurrences (interquartile range, 1–12) in the ANBP group, five occurrences (3–29) in the BN group, and no occurrences in the ANR group. The occurrence of hypoglycemia was statistically significantly higher in the BN group than in the ANR group (*P* = 0.013). There was a statistically significant difference in the proportion of patients with hypoglycemia in the three groups (*P* = 0.005). In this study, none of the participants reported subjective hypoglycemic symptoms.

**Fig. 2:**
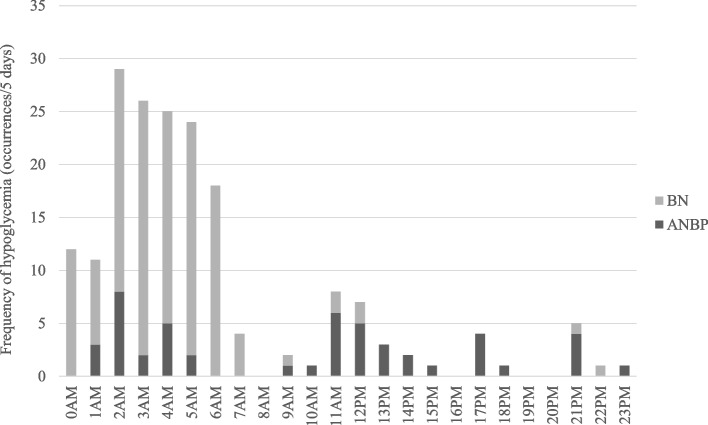
Time and frequency of hypoglycemia occurrence during a 5-day monitoring period. Legend: Hypoglycemia in the BN group (*n* = 5) was observed most frequently between 2 and 6 AM, while it was observed throughout the day in the ANBP group (*n* = 7). BN: bulimia nervosa; ANBP: anorexia nervosa, binge eating/purging subtype

Figure 2 shows the time and frequency of hypoglycemia occurrence during the five-day monitoring period. The overall frequency of hypoglycemia was highest between 2 and 6 AM, representing 66% of the total occurrence. In the BN group, the frequency of hypoglycemia was highest between 2 and 6 AM, while hypoglycemia was observed throughout the day in the ANBP group. No hypoglycemia was observed in the ANR group. Table 3 shows Spearman's correlation coefficient between the frequency of hypoglycemia and body weight and BMI in patients with hypoglycemia. No significant association was found between the frequency of hypoglycemia and body weight (*r* = 0.460, *P* = 0.190) or BMI (*r* = 0.421, *P* = 0.173).

**Table 3:** Correlation between the frequency of hypoglycemia and body weight and BMI in patients with hypoglycemia

	Weight (kg)	BMI (kg/m^2^)
The frequency of hypoglycemia (occurrences/5 days)	0.460	0.421

*Abbreviations*: *ANBP* Anorexia nervosa binge eating/purging, *BN* Bulimia nervosa

Presented by correlation coefficient (r) depending on the Spearman's rank correlation coefficient test

*n* = 12 (BN: *n* = 5, ANBP: *n* = 7)

The frequency of hyperglycemia during the 5-day monitoring period (Table 2) was as follows: one occurrence (interquartile range, 0–2) in the ANR group, one occurrence (0–5) in the BN group, and zero occurrences (0–1) in the ANBP group. No statistically significant associations were found between the three groups regarding the frequencies and proportions of people with hyperglycemia.

Table 4 shows the correlation between the frequency of binge eating and purging in the last 28 days and MAGE, hypoglycemia, and hyperglycemia. The MAGE index and the frequency of binge eating and vomiting showed a significant positive correlation. The frequency of hypoglycemia was significantly positively correlated with the frequency of binge eating.

**Table 4:** Correlation between the frequency of binge-eating and purging over the last 28 days and MAGE, hypoglycemia, and hyperglycemia

	Binge-eating (times/28 days)	Vomiting (times/28 days)	Laxative abuse (times/28 days)	Compulsive exercise (times/28 days)
MAGE (mg/dl)	0.596**	0.514*	-0.025	-0.069
The frequency of hypoglycemia (occurrences/5 days)	0.524*	0.313	0.116	0.146
The frequency of hyperglycemia (occurrences/5 days)	0.149	0.057	-0.229	-0.242

Presented by correlation coefficient (r) depending on the Spearman's rank correlation coefficient test

The data with statistically significant differences are shown by * *P* < 0.05 ** *P* < 0.01

*Abbreviations*: *MAGE* Mean amplitude of glycemic excursions

*n* = 18

**Fig. 3:**
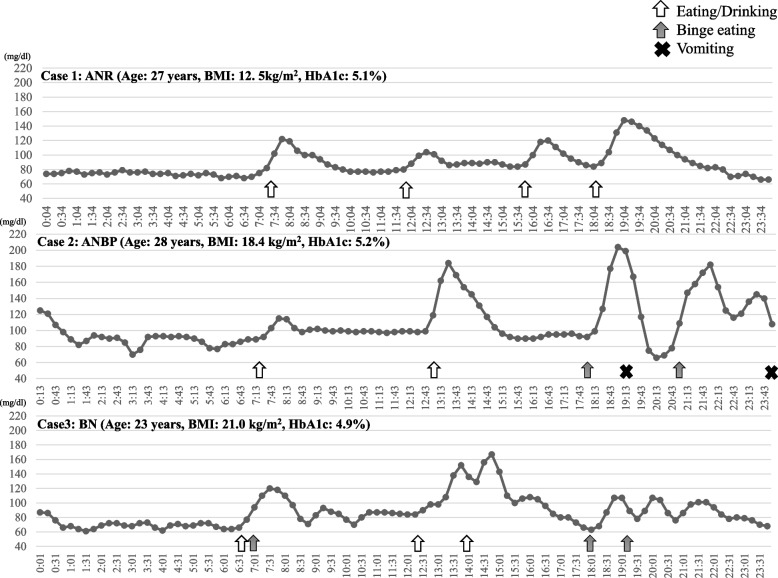
Glucose trends and eating behaviors in three eating disorder subtypes. Legend: (1) Patient with ANR: Age 27 years, BMI 12.5 kg/m^2^, HbA1c 5.1%, (2) Patient with ANBP: Age 28 years, BMI 18.4 kg/m^2^, HbA1c 5.2%, (3) Patient with BN: Age 23 years, BMI 21.0 kg/m^2^, HbA1c 4.9%. ANR: anorexia nervosa, restricting subtype; ANBP: anorexia nervosa, binge eating/purging subtype; BN: bulimia nervosa

Figure 3 shows the glucose trends and eating behaviors of three cases, all of whom had similar ages and HbA1c levels. The HbA1c level was normal in all three profiled cases. In case 1 (a patient with ANR), the glucose level increased from 84 mg/dL to 148 mg/dL by dinner and fell to pre-supper levels 3.5 h after dinner. In case 2 (a patient with ANBP), the glucose level increased by 112 mg/dL within 1 h after binge eating (from 92 mg/dL to 204 mg/dL). After vomiting, the glucose level decreased rapidly (by 133 mg/dL) in 1 h (i.e., from 199 mg/dL to 66 mg/dL). Case 3 (a patient with BN) had no occurrence of vomiting during the recording days. However, she had a more irregular dietary frequency, more irregular meal intervals, and more extreme glucose fluctuations than case 1 (a patient with ANR). Among these three cases, the bulimia patient presented the most frequent fluctuations in glucose level during the day; the lowest recorded glucose level was 61 mg/dL (at 1 AM).

## Discussion

To our knowledge, this is the first study to use an isCGM system to measure GV, hypoglycemia, and hyperglycemia in patients with eating disorders. Patients with eating disorders had high MAGE indices regardless of the presenting subtype. Patients with ANBP and BN tended to have higher MAGE indices and frequently developed hypoglycemia. In patients with BN, hypoglycemia developed frequently from midnight to early morning, while hypoglycemia developed throughout the day in patients with ANBP.

In the current study, the mean MAGE index in patients with eating disorders was higher than the average normal glucose tolerance regardless of eating disorder subtype [7–9]. Patients with ANBP and BN tended to have higher MAGE indices and frequently developed hypoglycemia. High MAGE indices in ANBP and BN patients suggested that eating behaviors such as binge eating and vomiting caused large glycemic fluctuations. A previous report showed that insulin and blood glucose levels measured in hospitalized patients with BN decreased rapidly after vomiting the test meal and that hypoglycemia due to vomiting could be associated with binge eating and purging cycles [16]. Moreover, this study found a decrease in the glucose level after vomiting.

Glucose levels were evaluated according to the type of disease using an isCGM system to measure glucose variability at 24 h. Several studies have reported that elevated MAGE indices are strongly associated with vascular endothelial dysfunction and have reported associations between the development of cardiovascular events and high MAGE indices in both diabetic and non-diabetic patients [17–19]. An evaluation using the previously reported cardio-ankle vascular index also reported early arteriosclerosis damage in a girl with AN [20]. Additional research is needed to investigate the relationship between the MAGE index and cardiovascular disease in patients with eating disorders.

Hypoglycemia was observed in 12 of 18 patients (67%), with three occurrences (interquartile range, 0–12) over the course of five days. Previous reports examining the development of hypoglycemia (glucose levels < 60 mg/dL) in hospitalized patients with eating disorders showed that 7.1% of 387 patients with ANR, 5.0% of 286 patients with ANBP,, and 6.5% of 251 patients with BN presented hypoglycemia on their admission blood tests [21]. In the current study, hypoglycemia was observed in five out of five patients with BN and seven out of nine patients with ANBP via 24-h CGM. Therefore, hypoglycemia was observed here more frequently than in the previous report [21]. This may be because 66% of hypoglycemic events in this study were observed from midnight to early morning, suggesting that hypoglycemia in patients with eating disorders is often overlooked if blood glucose levels are only collected during daytime hours.

The observed times and frequencies of hypoglycemia episodes that occur in patients with ANBP and BN (Fig. 2) suggest different patterns when comparing patients with ANBP and BN. Hypoglycemia was observed most often between 2 and 6 AM, while hypoglycemia was observed throughout the day in patients with ANBP. The reasons for nocturnal hypoglycemia in patients with BN could be as follows. First, excessive insulin secretion or delayed insulin secretion due to binge eating at night can lead to reactive hypoglycemia from night to early morning. Second, fasting biochemical hypoglycemia (FBH) may be related to hypoglycemia in patients with BN [22]. FBH is associated with women with low body mass. Many metabolic conditions in eating disorders, such as malnutrition or low food intake, low hepatic gluconeogenic capacity, and low glycogen storage, can contribute to the incidence of FBH. Third, bradycardia has been reported to occur in patients with BN due to hypervagal activity during sleep [23]. We consider that nocturnal hypoglycemia in patients with BN observed in this study may have changed insulin antagonist hormone metabolism due to hypervagal activity during sleep. Fourth, there may be unrecorded binge eating and vomiting at night, even in patients who keep daily records. However, the detailed mechanism of nocturnal hypoglycemia in patients with BN is unknown and requires further study.

In contrast, patients with ANBP developed hypoglycemia throughout the day. Starvation in patients with AN is believed to reduce hepatic glycogen content [24], and patients with ANBP have unstable dietary rhythms, such as binge eating, vomiting, and eating restrictions. These irregular dietary rhythms may lead to abnormal autonomic function and insulin secretion associated with hypoglycemia occurring throughout the day. As shown in case 2 (Fig. 3), the rapid decrease in glucose levels after vomiting in the profiled patient with ANBP may have been due to excessive insulin secretion during binge eating, because insulin continued to be secreted even after a large amount of gastric contents was expelled after vomiting. Thus, it is necessary to investigate changes in insulin secretion before and after binge eating and vomiting. One of the reasons hypoglycemia was not frequently observed in patients with ANR is that severe liver dysfunction was not observed in patients with AN who participated in this study. Previous reports have investigated the development of severe liver dysfunction and hypoglycemia in patients with ANR at hospital admission [25]. Our results on hypoglycemia in patients with eating disorders suggest the importance of developing different nutritional approaches tailored to subtypes of eating disorders to prevent hypoglycemia. First, nocturnal and early morning hypoglycemia in patients with BN may be associated with excessive or delayed insulin secretion after binge eating or FBH. Therefore, ways to prevent nocturnal and early morning hypoglycemia include nutritional treatment to avoid excessive intake of foods with a high glycemic index. Second, immediate glucose supplementation after vomiting may also be desirable for patients with ANBP. In general, it is recommended to take glucose (about 10 g) immediately when oral intake is possible during hypoglycemia in diabetic or non-diabetic patients. However, for example, the possibility of further reactive hypoglycemia due to inappropriate glucose intake cannot be ruled out. Further study is needed on an appropriate glucose supplementation method during hypoglycemia in patients with eating disorders.

Hyperglycemia was observed over the course of five days in patients with ANR who presented without binge eating (Table 2). Patients with ANR are generally reported to have reduced basal insulin secretion levels compared to healthy individuals and have statistically significantly delayed insulin secretion, as well as statistically significantly higher blood glucose levels 180 min after glucose loading compared to healthy individuals [26]. The gradual decrease in postprandial glucose in case 1 (a patient with ANR) was thought to be due to delayed insulin secretion (Fig. 3). Patients with ANR who restrict their diet do not require large amounts of insulin, although they could have reduced and delayed postprandial insulin secretion [26]. Delayed gastric emptying has also been reported in patients with AN, which can affect postprandial glucose metabolism [27]. Furthermore, impaired glucose tolerance is more likely to occur in underweight young women [28]. The glucose level of patients with ANR tends to increase after meals and may not easily fall after eating a meal.

However, three of the five patients with BN had hyperglycemia occurrences at least once during the 5-day monitoring period. Previous studies have reported that postprandial hyperglycemia is associated with eating and meal sequencing [29–31]. In the DSM-5, the definition of binge eating is 'eating, in a discrete period (e.g., within any two-hour period) an amount of food that is definitely larger than most people would eat in a similar period under similar circumstances’ [1]. In other words, patients who present with binge eating are considered to have a dietary habit of eating quickly that can be associated with a tendency to develop postprandial hyperglycemia. The frequency and intervals of meal intake in case 3 (a patient with BN) were irregular, showing the most frequent increases and decreases in glucose level among the three cases presented in Fig. 3. Furthermore, it has been suggested that visceral fat increases in patients with BN and weight-restored AN patients [32, 33]. Although no significant increase in HOMA-IR was observed in patients with BN in this study, further studies using CGM are considered necessary for postprandial hyperglycemia, which is difficult to evaluate with blood tests alone.

Our study has some limitations. First, the Free Style Libre Pro ® device used in this study has little associated data on its use in extremely underweight individuals; therefore, care must be taken when interpreting the results. In addition, because FreeStyle Libre Pro ® measures glucose levels in interstitial fluid, there are slight differences from actual blood glucose levels, with a mean absolute relative difference (MARD) of 11.4% [34]. Second, due to the small sample size, the statistical power of the current study might be inadequate, although no formal power calculations were conducted; therefore, differences between groups might be difficult to detect. Third, the patients with eating disorders enrolled in this study were all outpatients, and no severe patients (i.e., hospitalized psychiatric inpatients) were included. Finally, because this was not a longitudinal study, the causality of the evaluated relationships is unclear. Therefore, we recommend this topic be investigated more thoroughly in future highly powered prospective investigations.

## Conclusions

In the current study, we reported on GV, hyperglycemia, and hypoglycemia in patients with eating disorders using the isCGM system. Our findings suggest that outpatients with eating disorders have higher MAGE indices and that patients with ANBP and BN had a propensity to develop high MAGE and hypoglycemia. We also explored the frequent development of hypoglycemia from midnight to early morning in patients with BN, while hypoglycemia developed throughout the day in patients with ANBP. It has been suggested that eating behaviors such as binge eating and purging in patients with eating disorders are associated with GV and hypoglycemia. Thus, we used glucose monitoring to explore glycemic fluctuations in each evaluated subtype. Additional studies are needed to explore the relationship between glucose levels and eating behaviors in patients with eating disorders, as these findings inform prevention and intervention efforts to ameliorate the risk of severe morbidity and mortality occurring in patients with eating disorders.

## Data Availability

Data supporting the findings of this study are available upon request from the corresponding author. The data is not publicly available due to ethical restrictions.

## Abbreviations

AN: Anorexia Nervosa
ANR: Anorexia Nervosa Restricting
ANBP: Anorexia Nervosa Binge eating/Purging
BN: Bulimia Nervosa
DSM-5: The 5th edition of the Diagnostic and Statistical Manual of Mental Disorders
CGM: Continuous Glucose Monitoring
isCGM: Intermittently Scanned Continuous Glucose Monitoring
GV: Glycemic Variability
MAGE: Mean Amplitude of Glycemic Excursions
HOMA-IR: Homeostasis Model Assessment-Insulin Resistance
HOMA-β: Homeostasis Model Assessment-beta cell
FBH: Fasting Biochemical Hypoglycemia

## References

[1] American Psychiatric Association (2013). Diagnostic and Statistical Manual of Mental Disorders 5th ed: DSM-5.

[2] Herzog BD, Greenwood ND, Dorer JD, Flores TA, Ekeblad RE, Richards A (2000). Mortality in eating disorders: a descriptive study. Int J Eat Disord.

[3] Jáuregui-Garrido B, Jáuregui-Lobera I (2012). Sudden death in eating disorders. Vasc Health Risk Manag.

[4] Raevuori A, Suokas J, Haukka J, Gissler M, Linna M, Grainger M (2014). Highly increased risk of type 2 diabetes in patients with binge eating disorder and bulimia nervosa. Int J Eat Disord.

[5] Leelarathna L, Wilmot GE (2018). Flash forward: a review of flash glucose monitoring. Diabet Med.

[6] Suh S, Kim HJ (2015). Glycemic variability: how do we measure it and why is it important?. Diabetes Metab J.

[7] Chen T, Xu F, Su JB, Wang XQ, Chen JF, Wu G (2013). Glycemic variability in relation to oral disposition index in the subjects with different stages of glucose tolerance. Diabetol Metab Syndr.

[8] Hill RN, Oliver SN, Choudhary P, Levy CJ, Hindmarsh P, Matthews RD (2011). Normal reference range for mean tissue glucose and glycemic variability derived from continuous glucose monitoring for subjects without diabetes in different ethnic groups. Diabetes Technol Ther.

[9] Zhou J, Li H, Ran X, Yang W, Li Q, Peng Y (2011). Establishment of normal reference ranges for glycemic variability in Chinese subjects using continuous glucose monitoring. Med Sci Monit.

[10] Ravi R, Balasubramaniam V, Kuppusamy G, Ponnusankar S (2021). Current concepts and clinical importance of glycemic variability. Diabetes Metab Syndr.

[11] University of Oxford: https://www.phc.ox.ac.uk/research/resources/easygv Accessed 22 Oct 2021

[12] Meneilly GS, Cheung E, Tuokko H (1994). Altered responses to hypoglycemia of healthy elderly people. J Clin Endocrinol Metab.

[13] Miller CD, Phillips LS, Ziemer DC, Gallina DL, Cook CB, El-Kebbi IM (2001). Hypoglycemia in patients with type 2 diabetes mellitus. Arch Intern Med.

[14] Matthews DR, Hosker JP, Rudenski AS, Naylor BA, Treacher DF, Turner RC (1985). Homeostasis model assessment: insulin resistance and beta-cell function from fasting plasma glucose and insulin concentrations in man. Diabetologia.

[15] Matthews DR, Hosket JP, Rudenski AS, Naylor BA, Treacher DF, Turner RC (1985). Homeostasis model assessment: insulin resistance and beta-cell function from fasting plasma glucose and insulin concentrations in man. Diabetologia.

[16] Johnson GW, Jarrell PM, Chupurdia MK, Williamson AD (1994). Repeated binge/purge cycles in bulimia nervosa: role of glucose and insulin. Int J Eat Disord.

[17] Su G, Mi SH, Li Z, Tao H, Yang HX, Zheng H (2013). Prognostic value of early in-hospital glycemic excursion in elderly patients with acute myocardial infarction. Cardiovasc Diabetol.

[18] Su G, Mi SH, Tao H, Li Z, Yang HX, Zheng H (2013). Impact of admission glycemic variability, glucose, and glycosylated hemoglobin on major adverse cardiac events after acute myocardial infarction. Diabetes Care.

[19] Akasaka T, Sueta D, Tabata N, Takashio S, Yamamoto E, Izumiya Y (2017). Effects of the mean amplitude of glycemic excursions and vascular endothelial dysfunction on cardiovascular events in nondiabetic patients with coronary artery disease. J Am Heart Assoc.

[20] Tonhajzerova I, Mestanikova A, Jurko A, Grendar M, Langer P, Ondrejka I, Jurko T, Hrtanek I, Cesnekova D, Mestanik M (2020). Arterial stiffness and haemodynamic regulation in adolescent anorexia nervosa versus obesity. Appl Physiol Nutr Metab.

[21] Mehler PS, Blalock VD, Walden K, Kaur S, McBride J, Walsh K (2018). Medical findings in 1,026 consecutive adult inpatient-residential eating disordered patients. Int J Eat Disord.

[22] Tanaka K, Higuchi R, Mizusawa K, Nakamura T, Nakajima K (2021). Fasting biochemical hypoglycemia and related-factors in non-diabetic population: Kanagawa Investigation of Total Check-up Data from National Database-8. World J Diabetes.

[23] Kennedy SH, Heslegrave RJ (1989). Cardiac regulation in bulimia nervosa. J Psychiatr Res.

[24] Rosen E, Bakshi N, Watters A, Rosen RH, Mehler SP (2017). Hepatic complications of anorexia nervosa. Dig Dis Sci.

[25] Rosen E, Sabel AL, Brinton JT, Catanach B, Gaudiani JL, Mehler PS (2016). Liver dysfunction in patients with severe anorexia nervosa. Int J Eat Disord.

[26] Tanaka M, Tatebe Y, Nakahara T, Yasuhara D, Sagiyama K, Muranaga T (2003). Eating pattern and the effect of oral glucose on ghrelin and insulin secretion in patients with anorexia nervosa. Clin Endocrinol.

[27] Heruc GA, Little TJ, Kohn MR, Madden S, Clarke SD, Horowitz M (2018). Effects of starvation and short-term refeeding on gastric emptying and postprandial blood glucose regulation in adolescent girls with anorexia nervosa. Am J Physiol Endocrinol Metab.

[28] Sato M, Tamura Y, Nakagata T, Someya Y, Kaga H, Yamasaki N (2021). Prevalence and features of impaired glucose tolerance in young underweight Japanese women. J Clin Endocrinol Metab.

[29] Kuwata H, Iwasaki M, Shimizu S, Minami K, Maeda H, Seino S (2016). Meal sequence and glucose excursion, gastric emptying and incretin secretion in type 2 diabetes: a randomised, controlled crossover, exploratory trial. Diabetologia.

[30] Imai S, Fukui M, Ozasa N, Ozeki T, Kurokawa M, Komatsu T (2013). Eating vegetables before carbohydrates improves postprandial glucose excursions. Diabet Med.

[31] Saito Y, Kajiyama S, Nitta A, Miyawaki T, Matsumoto S, Ozasa N (2020). Eating fast has a significant impact on glycemic excursion in healthy women: randomized controlled cross-over trial. Nutrients.

[32] Kim Y, Hildebrandt T, Mayer LES (2019). Differential glucose metabolism in weight restored women with anorexia nervosa. Psychoneuroendocrinology.

[33] Ludescher B, Leitlein G, Schaefer JE, Vanhoeffen S, Baar S, Machann J (2009). Changes of body composition in bulimia nervosa: increased visceral fat and adrenal gland size. Psychosom Med.

[34] Bailey T, Bode BW, Christiansen MP, Klaff LJ, Alva S (2015). The performance and usability of a factory-calibrated flash glucose monitoring system. Diabetes Technol Ther.

